# HIV Epidemic among Young Thai Men, 1991–2000

**DOI:** 10.3201/eid0907.020653

**Published:** 2003-07

**Authors:** Kalyanee Torugsa, Scott Anderson, Nucharee Thongsen, Narongrid Sirisopana, Achara Jugsudee, Pitak Junlananto, Sorachai Nitayaphan, Suebpong Sangkharomya, Arthur E. Brown

**Affiliations:** * Armed Forces Research Institute of Medical Sciences, Bangkok, Thailand; † State University of New York at Cortland, Cortland, New York, USA; ‡ Army Institute of Pathology, Bangkok, Thailand

**Keywords:** human immunodeficiency virus, epidemiology, Thailand, geographic information systems, dispatch

## Abstract

Characterization of the HIV epidemic in Thailand has benefited from the systematic testing of young men upon entry into the military. These data, which have shown that public health measures can reverse an HIV epidemic, have been reanalyzed with current geographic information systems methods. The resulting maps, thus far, are the best means of visualizing the geography of the dynamic HIV epidemic in Thailand.

In few nations is the HIV epidemic characterized as well as it is in Thailand. As elsewhere, sentinel surveillance of high-risk populations is performed and more general populations are partially assessed by HIV testing of blood donors and pregnant women. In addition, Thailand has benefited from another measure to assess the HIV epidemic in the general population. Since very early in the epidemic, young men have been tested for HIV at the time they entered into military service. Military medicine and public health leaders recognized that, in addition to using this information to benefit individual men, maintaining this information in a confidential database linked to area of residence would allow monitoring of national demographic trends in the epidemic. Data generated in this way have provided key evidence that public health policy has reversed the trend of increasing HIV infections (*1*), preventing an estimated 200,000 HIV infections by 2000 (*2*).

The HIV prevalence data on young Thai men is provided to public health policy makers on a regular basis. It has also been published in the scientific literature (*3*,*4*) with data presented that uses provinces as the scale of analysis. More recently, this unique dataset has been analyzed by using geographic information systems (GIS). GIS methods allow enhanced visualization of the trends, both geographic and chronologic, within this well-documented HIV epidemic. Using a GIS approach, we present the Royal Thai Army dataset, expanded to include the decade of 1991–2000 and data precision refined to the district level.

## Methods

The methods of recruiting young men into the Thai military have been described previously (*3*,*4*). Approximately 50,000–60,000 men, mostly 21 years of age, are selected each year by lottery in their home province (the province in which they are listed on the house registration). The system produces a representative national sampling of Thai men. Because of their young age, HIV prevalence in these annual recruit classes may serve as a proxy for HIV incidence. Induction into the military occurs in May (M) and November (N) of each year. At the time when blood is collected, recruits provide information about the location of their main residence (including province and district) during the previous 2 years (*3*). Although the actual locations where infections occurred are unknown, residential data enables analysis of the association between HIV prevalence and this key geographic marker.

To refine the HIV prevalence analyses, geographic localization uses districts as the first administrative subunit of provinces. When data were analyzed by using annual classes grouped at the district level, calculations of the percentage testing positive for HIV were statistically unreliable because the number of men tested in some rural districts was so small. Therefore, we merged data in two ways to decrease variability attributable to the small sample size of the prevalence figures: classes were combined across time, and districts were combined across space. Sixteen classes of men recruited from 1991 to 2000 were combined temporally into four larger classes, each representing discrete 2-year periods: 1) N91, M92, N92, M93; 2) N94, M95, N95, M96; 3) N96, M97, N97, M98; and 4) N98, M99, N99, M00. Data on classes recruited from the M91, N93, M94, and N00 lotteries were not available for analysis (M91, before full implementation; N93 and M94, protocol under revision; N00, completed after dataset closed). However, even after combining classes into these 2-year periods, a number of districts still had numbers too low for statistical reliability.

We also merged some districts with neighboring districts so that each had a minimum denominator of 20 in the HIV prevalence calculation. Numbers of >20 persons provide minimal, but acceptable, reliability in the percentage-positive calculations. Districts with <20 were combined with other districts according to the following protocol, following a sequence of priorities: we combined districts if they were in the same province, had historic connections (formerly part of single larger district), had similarly small numbers tested, had similar demographics, and had similar topography or other geographic features.

For the GIS analysis, data tables provided in Excel files (Microsoft Corp., Redmond, WA) by the Armed Forces Research Institute of Medical Sciences were joined to district-level GIS maps obtained from the Thai Environmental Institute and National Statistical Office. We used Arcview 3.2a software (ESRI, Redlands, CA) to create dot density and choropleth maps. (A choropleth map uses shades or colors to demonstrate the geographic distribution of a range of values.)

## Results and Discussion

**Figure 1:**
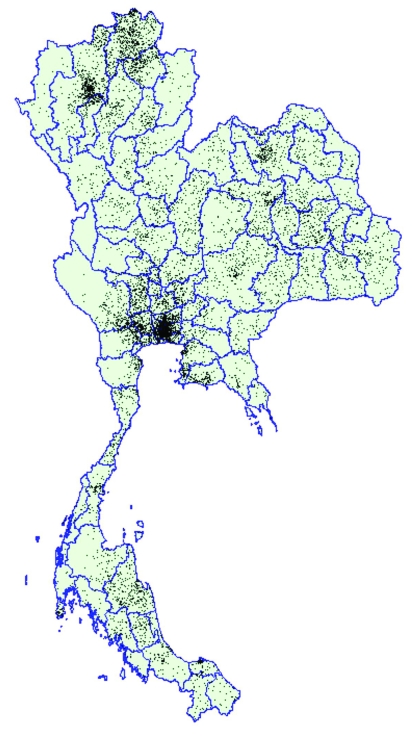
Dot density map of young men who tested positive for HIV at time of entry into the Royal Thai Army, Thailand, November 1991–May 2000. Each dot represents one man. Location of dots based on recruit’s residence during the previous 2 years. Data on recruits entering in November 1993 and May 1994 are not available.

Figure 1 provides visual evidence of the geographic distribution of the epidemic. Each of the 10,043 dots represents a recruit who tested positive for HIV infection (positive for HIV-1 by enzyme immunoassay and Western blot) among the 442,923 recruits tested. Arcview software uses an algorithm that allows the random placement of dots within a geographic area (in this case a district). By creating these dots at the district level but presenting them at the province level, we show the vivid geographic pattern of infection while illustrating the human impact of the epidemic. The dot distribution does not distinguish between population density and HIV prevalence. Nevertheless, these absolute numbers provide important information regarding the potential HIV-related impact on the healthcare system and various other social structures.

**Figure 2:**
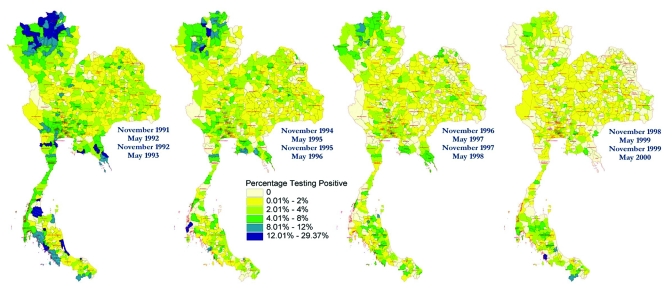
Choropleth maps of HIV prevalence in four classes of young men at time of entry into the Royal Thai Army, Thailand, 1991–2000. Location determined by residence during the previous 2 years. Prevalence is stratified by color and localized to district or group of districts so that calculations are based on >20 men.

The evolution of the HIV epidemic over the course of the decade is seen in the four maps shown in Figure 2. These maps use gradients of color shading to distinguish the percentage of men who tested positive for HIV during each 2-year period. The initial high prevalence of HIV in men from the upper north region is illustrated, as is the decline in prevalence over time (which likely occurred as a result of public health interventions). The relative persistence of HIV prevalence in some districts in the far south of the country is also shown, suggesting that locale-specific features of the epidemic need to be well understood in that area, potentially leading to changes in public health interventions.

Previous analyses conducted at the province level identified regional variation quite clearly, but at a less detailed level (*3*,*4*). The maps from our study indicate considerable variation even to the district level of analysis, which suggests important local factors may be at work in determining rates of transmission. The HIV epidemic in Thailand seems to have largely missed some districts and devastated others, even within the same province. Chiang Mai and Chiang Rai Provinces are examples of this phenomenon. These maps also show that, although the growth of the epidemic has slowed in most parts of the country, the epidemic has not decreased in some districts in south Thailand. Choropleth maps best demonstrate the changing prevalence of HIV among recruits from each district. The dot map best demonstrates the intensity of the epidemic within and across provinces. Both types of imagery are required to describe and understand the epidemic and to suggest locales in which public health initiatives are needed. In areas where the intensity of the epidemic is highest, health and social services may require additional resources to serve persons with the disease. In areas where the prevalence rates are highest or are increasing, socially and culturally appropriate interventions may be needed to strengthen and focus HIV programs.

These GIS maps are the most accurate maps available to date and are built upon the unique HIV prevalence datasets collected over a decade by the Royal Thai Army. GIS mapping at this descriptive level enhances visualization of these data (*5*,*6*). More sophisticated methods of multivariate GIS visualization could enhance this analysis even more but would require the Royal Thai Army to assess recruits at induction on other variables, especially risk factors, or to use recruits’ home addresses rather than districts or provinces of residence. Nonetheless, changes in time and place of chronic viral infection can be added to the recognized areas of GIS application in epidemiology, transmission of vector-borne and water-borne diseases, and environmental health (*7*). The maps also illustrate the point that political borders may have no relationship to epidemiologic boundaries. Differences in HIV prevalence often occur, not only between regions and provinces but also within provinces and across provincial borders. When an epidemic can be visualized at this level of detail and accuracy, researchers can formulate and address questions regarding the bases of these differences. At the same time, policy makers can better direct intervention strategies and more finely assess the outcomes of these interventions. Coordination of data collection and joint GIS analysis by neighboring countries would enhance regional understanding of the emergence and spread of HIV epidemics and monitoring of control programs.
